# Usual Intake of Key Minerals among Children in the Second Year of Life, NHANES 2003–2012

**DOI:** 10.3390/nu8080468

**Published:** 2016-07-30

**Authors:** Heather C. Hamner, Cria G. Perrine, Kelley S. Scanlon

**Affiliations:** National Center for Chronic Disease Prevention and Health Promotion, Centers for Disease Control and Prevention (CDC), Atlanta, GA 30341, USA; hgk3@cdc.gov (C.G.P.); kelley.scanlon@fns.usda.gov (K.S.S.)

**Keywords:** iron, calcium, zinc, young children, usual nutrient intake, NHANES

## Abstract

Iron, calcium, and zinc are important nutrients for the young, developing child. This study describes the usual intake of iron, calcium, and zinc among US children in the second year of life using two days of dietary intake data from the National Health and Nutrition Examination Survey 2003–2012. Estimates were calculated using PC-SIDE to account for within and between person variation. Mean usual iron, calcium, and zinc intakes were 9.5 mg/day, 1046 mg/day, and 7.1 mg/day, respectively. Over a quarter of children had usual iron intakes less than the Recommended Dietary Allowance (RDA) (26.1%). Eleven percent of children had usual calcium intakes below the RDA and over half of children had usual intakes of zinc that exceeded the tolerable upper intake level (UL). Two percent or less had usual intakes below the Estimated Average Requirement (EAR) for iron, calcium, and zinc. Our findings suggest that during 2003–2012, one in four children and one in ten children had usual intakes below the RDA for iron and calcium, respectively. Children who are not meeting their nutrient requirements could be at increased risk for developing deficiencies such as iron deficiency or could lead to a shortage in adequate nutrients required for growth and development. One in every two children is exceeding the UL for zinc, but the interpretation of these estimates should be done with caution given the limited data on adverse health outcomes. Continued monitoring of zinc intake and further assessment for the potential of adverse health outcomes associated with high zinc intakes may be needed.

## 1. Introduction

Iron, calcium, and zinc are key minerals needed to ensure optimal cognitive development [1,2], bone health [3], and growth [1]. For young children, the American Academy of Pediatrics (AAP) has identified iron and zinc as critical nutrients—especially for children who are exclusively breastfed and are transitioning to the introduction of complementary foods [4]. Although the majority of children 12–23 months of age have transitioned to solid foods, this time period is still an important period of physical and cognitive development and adequate nutrient intakes are needed [5]. Iron, calcium, and zinc are needed throughout early childhood. Iron is important for optimal cognitive development [1]; calcium is critical in the development of bones and teeth and can be especially important during growth spurts [3], and zinc is important in growth [1]. Ensuring adequate intake of iron, calcium, and zinc can help reduce the risk of developing severe deficiencies such as iron deficiency anemia [1,2] or impaired growth, such as rickets [1,3]. Conversely, nutrient intakes exceeding cut points such as the tolerable upper intake level (UL) could lead to adverse consequences; however, limited data are available on functional outcomes for young children with regard to higher intake values [1,3].

Nationally representative data on the nutrient intake for this age group as well as the proportions who are meeting Dietary Reference Intakes, such as the Estimated Average Requirement (EAR), the Recommended Dietary Allowance (RDA), and those exceeding the UL, are lacking. Nutrition intake estimates among this age group can inform clinicians about key nutrient intakes during a critical growth period, as well as provide a basis for population level estimates that can support efforts, such as the United States Department of Agriculture (USDA)/Health and Human Services (HHS) Dietary Guidance Development Project for Birth to 24 Months and Pregnancy (B24/P) [6]. We focus this analysis on three key minerals needed for growth and development (iron, calcium, and zinc) for children 12–23 months of age and present the proportion meeting the EAR, RDA, and those exceeding the UL.

## 2. Materials and Methods 

### 2.1. National Health and Nutrition Examination Survey

NHANES is an ongoing nationally representative survey of the noninstitutionalized civilian US population [7]. The survey is conducted using a stratified multistage probability design. Data from NHANES are released in 2-year cycles. This analysis includes data from 2003 to 2012. Survey respondents participate in a household interview in which participants are asked a variety of questions including information on demographics and health-related questions and a physical examination in which participants undergo a medical exam and participate in a dietary interview. Analyses reported by race/ethnicity were restricted to non-Hispanic white, non-Hispanic black, and Mexican American respondents because of the small number of individuals of other racial and ethnic groups; however, all race/ethnicities are included in analyses not stratified by race/ethnicity. We limited our analyses to children who were aged 12–23 months at the time of the physical examination. All participants in NHANES provide written informed consent or by proxy for those who are under 7 years of age. The National Center for Health Statistics Research Ethics Review Board provided the following protocol approval numbers for the presented survey years: Protocol #98-12 (NHANES 1999–2004), Protocol #2005-06 (NHANES 2005–2006), Continuation of Protocol #2005-06 (NHANES 2007–2010), and Protocol #2011-17 (NHANES 2011–2012).

### 2.2. Nutrient Intake

Usual nutrient intake (calories, iron, calcium, and zinc) was estimated using two 24 h dietary recall questionnaires. The first dietary recall was conducted in-person and the second dietary recall was conducted 3–10 days later via telephone. Dietary interviews for children less than 6 years of age were conducted using a proxy (i.e., a parent) who was most familiar with the child’s dietary intake [8]. The USDA Food and Nutrient Database for Dietary Studies was used to determine the nutrient amount for foods that are reported in NHANES 2003–2012 [9,10,11,12,13]. Total nutrient intake for day one and day two of the 24 h dietary recall were used in analyses. 

### 2.3. Analytic Sample

There were a total of 1534 children aged 12–23 months at the time of the physical examination from 2003 to 2012 and were eligible to complete a dietary recall. Children were excluded if they reported consuming any breast milk on either day one or day two of the dietary interview (*n* = 94) because nutrient intakes from breast milk were not available and therefore, total nutrient intake could not be calculated. Additionally, children who did not have a dietary intake record for day one and day two, or who had a dietary record that was coded as not reliable, were excluded (*n* = 318), leaving a final sample size of 1122 (78% of the eligible sample who did not consume any breast milk). 

### 2.4. Covariates 

Information on age, race/ethnicity, and income to poverty ratio were obtained through the household interview questionnaire. Race/ethnicity was based on respondents’/parental answers to questions on race and Hispanic origin. The income to poverty ratio, a ratio of family income to poverty guidelines, was based on the family’s reported household income. The income to poverty ratio was split into three categories: (1) income to poverty ratios <1.85; (2) income to poverty ratios between 1.85 and less than 3.5; and (3) income to poverty ratios ≥3.5. These income to poverty ratios correspond to income eligibility cut-offs used in the United States Department of Agriculture Women, Infants, and Children Program.

### 2.5. Statistical Analyses

Using data from the two 24-h dietary recalls, the usual intakes of total caloric intake (for reference), iron, calcium, and zinc for children 12–23 months of age were estimated using software developed by Iowa State University, PC-SIDE version 1.02 (Iowa State University, Ames, IA, USA) and within-person variation of nutrient intake were accounted for across days. In addition, the proportion of children below two specific cut-points, i.e., estimated average requirement (EAR) and recommended dietary allowance (RDA), and the proportion above the cut-points for tolerable upper intake level (UL) for each mineral were assessed. The EAR is the average daily nutrient intake estimated to meet the needs of half the healthy children of this age; whereas, the RDA is the average daily nutrient intake estimated to meet the needs of nearly all healthy children of this age [14]. The UL is the highest average daily intake likely to pose no adverse health effects [14]. Usual intakes were adjusted for the intake day of the week and interview method (in person vs. telephone). Estimates of usual intake and proportions were calculated by sex, race/ethnicity, and income to poverty ratio. Since usual intakes cannot be negative, any estimates that were negative (i.e., lower bound for 95% Confidence Interval (CIs)) were truncated at zero. Additionally, if a cut-point fell on the distribution of intakes such that no individual was included, these values did not have a standard error; thus, a zero value was given and no 95% CIs were provided. 

SPSS Complex Samples Design version 23.0 (SPSS Inc., Chicago, IL, USA) was used to account for the survey design and calculate frequencies and Chi-square tests. All analyses were conducted using 10-year dietary weights calculated from day two dietary weights for the period 2003–2012, as recommended by the National Center for Health Statistics, Centers for Disease Control and Prevention [15,16]. For analyses conducted with PC-SIDE, standard errors were calculated using a set of 150 Jackknife replicate weights calculated using the 10-year dietary weights. T-tests were calculated to assess differences in mean usual intakes; statistical significance defined as *p* < 0.05.

## 3. Results

A total of 1122 children aged 12–23 months of age were included in the analysis. Approximately half of children were male (52%) (weighted percent). Over half (53.6%) of children were non-Hispanic white, 14.4% were non-Hispanic black, and 17.8% were Mexican American (weighted percent). Among the analytic sample, 49.3% reported an income to poverty ratio <1.85 (weighted percent). 

Among children 12–23 months of age, the mean usual caloric intake was 1264 kcal/day (95% CI: 1225, 1302). Caloric intake did not differ by sex or income to poverty ratio, but non-Hispanic black children had significantly higher usual caloric intake than either non-Hispanic white children or Mexican American children (1350 kcal/day, 1267 kcal/day, and 1218 kcal/day, respectively) (*p* < 0.05). Mean usual iron intake was 9.5 mg/day (Table 1). Compared to non-Hispanic white and non-Hispanic black children, Mexican-American children had significantly lower reported mean usual iron intake (9.6 mg, 10.2 mg, and 8.5 mg, respectively) (*p* < 0.05). Mean usual calcium intake was 1046 mg/day; no differences were observed by sex, race/ethnicity, or poverty status. Mean usual zinc intake was 7.1 mg/day; girls had significantly lower zinc intake compared to boys (6.9 mg vs. 7.3 mg, respectively) (*p* < 0.05). 

Less than 1% of children had usual iron intakes below the EAR (3 mg/day) [1]; however, 26.1% had usual intakes below the RDA (7 mg/day) [1]. Mexican American children had significantly higher proportions below the RDA for iron compared to non-Hispanic white and non-Hispanic black children (36.4%, 24.3%, and 18.7%, respectively) (*p* < 0.05). No children had usual iron intakes exceeding the UL (40 mg/day) [1]. 

Two percent of children 12–23 months had usual calcium intake below the EAR (500 mg/day) [3] and 11.2% had usual calcium intakes below the RDA (700 mg/day) [3] (Table 2). Non-Hispanic black children and children with an income to poverty ratio <1.85 had significantly higher proportions below the RDA for calcium (*p* < 0.05). There were no children 12–23 months with usual calcium intakes that exceeded the UL (2500 mg/day) [3]. 

Less than 1% of children had usual zinc intakes below either the EAR (2.5 mg/day) [1] or the RDA (3 mg/day) [1]. Over 50% of children had usual zinc intakes that exceeded the UL (7 mg/day) [1]; no differences by sex, race/ethnicity, or income to poverty ratio were observed. 

## 4. Discussion

Our analyses presented nationally representative usual mean intake for children 12–23 months of age on key minerals needed for healthy growth and development [1,3]. Our findings indicate that one in four children and one in ten children 12–23 months of age are not consuming enough iron and calcium to meet current RDA recommendations, respectively. However, one in two children 12–23 months of age are exceeding the UL for zinc. 

Our findings, presented here, are similar to what was reported in the 2008 Feeding Infants and Toddler Study (FITS) [17], a cross-sectional consumer panel survey weighted to be nationally representative. For example, FITS reported usual intake of zinc 7.2 mg/day and our analysis indicated a usual intake of 7.1 mg/day. However, FITS data had slightly lower usual intakes of calories and calcium and higher usual intakes of iron as compared to our results using NHANES (FITS: 1141 kcal/day; 892 mg/day calcium; 10.3 mg/day iron; NHANES: 1264 kcal/day; 1046 mg/day calcium; 9.5 mg/day iron) [17]. The FITS 2008 findings assessed both the proportion below the EAR and the proportion exceeding the UL and found similar results as those presented here. Although FITS is considered nationally representative, it may not be truly representative if the consumer panel used as the sampling frame is not representative of the US population. Although the surveys had slightly different estimates, both came to similar conclusions in regard to nutrient intake of key minerals for children in the second year of life (12–23 months of age). 

Our analysis expanded on the FITS assessment by looking at the proportion of children 12–23 months of age with usual intakes below the RDA. One in every four children, and one in every three children who was Mexican American, reported having an iron intake below the RDA and the intake value recommended by the AAP [1,2]. A recent study by Grimes et al. reported the top food sources of iron for this age group and found ready-to-eat cereals, baby foods, breads, rolls and tortillas, mixed dishes—grains, and cooked cereals were responsible for half of a child’s total daily iron intake [18]. These sources are a combination of multiple foods and could represent fortified sources (i.e., ready-to-eat cereals), as well as heme-rich sources (i.e., baby foods with meat) or non-heme sources (i.e., baby foods with fruit and/or vegetables). Adequate intake of iron can help reduce the likelihood of developing iron deficiency and iron deficiency anemia [2]. According to data from NHANES 2007–2010, 13.5% of children 1–2 years of age were considered iron deficient [19]. Given the importance of iron in optimal cognitive development at this age [1,2], the reported estimates of iron deficiency [19] and iron intake suggest the need to ensure young children are consuming adequate iron and to continue monitoring iron status. 

Calcium has also been identified as a key mineral to ensuring adequate growth and development of young children, especially for bone health [3]. With the development of the 2011 Institute of Medicine report on calcium and vitamin D, there are now EARs and RDAs for calcium for children 1–3 years of age; these values were not available in the previous 1997 IOM report for calcium and vitamin D [3,20]. Our data indicate that although very few children 12–23 months of age have a usual calcium intake below the EAR, one in ten, and almost one in five non-Hispanic black children (17.4%), have an intake below the RDA. Milk is the main food group that contributes to calcium intake for this age group with over 50% of daily intake of calcium coming from milk [18]. However, trends in beverage consumption indicate a significant decline in milk consumption among 1 year olds (3.8% decline) and an increase in 100% fruit juice consumption (21.9% increase) from 1988–1994 to 2001–2006 [21]. Continued support for ensuring adequate calcium intake through sources like milk is important. 

Ensuring adequate zinc intake for young children, especially during the transition from breast milk to complementary foods, has been one of the cornerstones of AAP recommendations [4]. However, our data indicate that over half of children 12–23 months have usual zinc intakes that exceed the UL, which is similar to what Butte et al. reported using FITS [17]. Milk and ready-to-eat cereals were the top two food sources contributing 39.1% of total zinc intake among children 12–23 months of age [18]. Ready-to-eat cereals may be fortified with zinc and could be one of the reasons for higher zinc intakes in this age group. Data were not available on children 1–3 years of age to set a UL value for zinc for this age group[1]; however, data were available from one study among 68 infants 0–6 months of age receiving infant formula with 5.8 mg zinc/L of formula for six months found no adverse effects [22]. Using this study as a basis, the UL value for zinc among children 1–3 years of age was extrapolated and then adjusted for body weight [1]. Two case reports of children receiving ≥16 mg of zinc for ≥6 months developed a copper-induced anemia [23,24]; however, evidence of zinc toxicity in young children is not often reported [25,26] and may not be a concern at the population level. Additionally, the potential that the zinc UL for children is too low has been raised [27,28]. Therefore, interpreting whether the proportion of children above the UL for zinc is of concern should be done with caution since limited data are available supporting population-level indications of adverse health outcomes associated with high intake and the relevance of the current UL value has been questioned. Continued monitoring of zinc intake and the potential for adverse health outcomes could be warranted. 

This analysis provides pediatricians, other health care providers, and public health practitioners with evidence on the nutritional intake of young children, specifically for key minerals needed for healthy growth and development. Compared with developing countries, children in the United States may not be considered as at risk for specific nutritional deficiencies; however, this analysis indicates that for specific minerals, such as iron, there may be a need for a renewed focus on ensuring children are consuming adequate nutrients. A further assessment of nationally representative data on the nutritional status of young children using biomarkers could help provide context to national level policies and recommendations for foods and food groups to encourage. Biomarkers, with the exception of iron, on this age group are not routinely collected through surveys, such as NHANES, but specific biomarkers may need to be considered given the assessment of current intake and the development of the B24/P Guidance. 

This study is subject to several limitations. First, we combined multiple survey years. Although this provides a larger sample size and smaller standard errors, the data span a period of ten years. Sociodemographic characteristics did not differ by survey year; however, we did find that usual caloric intake was significantly higher in 2003–2004. We did not correct for this because we were not assessing trends over time and recommendations, such as the EAR, RDA and UL are set values and are not dependent on total caloric intake. Second, we did not include any nutrient intake coming from dietary supplements, which could contribute to mineral intake. This decision was based on a change in methodology for reporting supplement intake during the survey years included in our analysis. When we assessed the frequency of supplement use in a subset of the population with similar supplement intake methodology, we concluded that the inclusion of dietary supplements would not change the overall interpretation of our results. Specifically, we found that among children 12–23 months of age surveyed in 2007–2012, 13.3% reported consuming any dietary supplement on day 1 Lastly, we limited our analyses to children not reporting the consumption of breast milk during the second year of life on day one or day two of the dietary intake recall. This was done because nutrient intakes were not reported for children who reported consuming any breast milk. A total of 94 children reported some consumption of breastmilk (6% of the original sample). As a result, these findings may not be generalizable to children consuming breast milk during the second year of life. There were also several strengths of this study. First, NHANES is a nationally representative study. Second, usual dietary intake assessment was possible because two 24-h dietary recalls were collected and nutrients examined were consumed on a daily basis and were not episodic. This allowed for the estimation of the proportions of the population at specific cut-points (i.e., EAR, RDA, and UL). Lastly, because we combined survey years, we were able to have adequate sample size to stratify by different sociodemographic factors. 

## 5. Conclusions 

During 2003–2012, one in every four and one in every ten children 12–23 months of age is not meeting the recommended iron and calcium intake, respectively. Efforts to ensure children are consuming optimal amounts of both iron and calcium are important for their growth and development. One in every two children 12–23 months of age is exceeding the UL for zinc, but the interpretation of these estimates should be done with caution given the limited data on adverse health outcomes. 

## Figures and Tables

**Table 1:** Mean usual iron, calcium, and zinc intake for children age 12 to 23 months ^1,2^ by select demographic characteristics, NHANES 2003–2012.

	Mean (95% Confidence Interval)		
	Iron (mg)	Calcium (mg)	Zinc (mg)
**Total** (*n* = 1122)	9.5 (9.0, 10.0)	1046 (1002, 1090)	7.1 (6.9, 7.4)
**Sex**			
Male (*n* = 574)	9.8 (9.1, 10.5)	1050 (992, 1108)	7.3 (7.0, 7.7) ^a^
Female (*n* = 548)	9.1 (8.5, 9.7)	1041 (985, 1097)	6.9 (6.7, 7.2) ^b^
**Race/ethnicity** ^3^			
Non-Hispanic white (*n* = 332)	9.6 (8.9, 10.4) ^a^	1055 (981, 1128)	7.2 (6.8, 7.6)
Non-Hispanic black (*n* = 261)	10.2 (9.4, 11.1) ^a^	983 (909, 1056)	7.2 (6.6, 7.7)
Mexican American (*n* = 357)	8.5 (8.0, 9.1) ^b^	1047 (993, 1100)	7.2 (6.8, 7.5)
**Poverty status**			
Income to poverty ratio <1.85 (*n* = 684)	9.4 (8.8, 9.9)	1020 (973, 1068)	7.2 (6.9, 7.5)
Income to poverty ratio 1.85 to <3.5 (*n* = 204)	9.2 (8.3, 10.1)	1056 (962, 1149)	7.0 (6.5, 7.5)
Income to poverty ratio ≥3.5 (*n* = 174)	9.8 (8.7, 10.9)	1095 (1011, 1180)	7.1 (6.6, 7.6)

^1^ Does not include any children who reported consuming breast milk on either day one or day two of the 24-h dietary recall; ^2^ age in months at time of exam in Medical Examination Center; ^3^ race/ethnicity subanalyses are limited to those individuals who report being either non-Hispanic white, non-Hispanic black, and Mexican American; values with superscript letters that differ are significantly different, *p*-value < 0.05; abbreviations: National Health and Nutrition Examination Survey (NHANES).

**Table 2:** Percent of children aged 12 to 23 months ^1,2^ not meeting recommendations for iron, calcium and zinc by select demographic characteristics, NHANES 2003–2012 ^3^.

	% (95% Confidence Interval)								
	Iron			Calcium			Zinc		
	% below EAR (3 mg/Day)	% below RDA (7 mg/Day)	% above UL (40 mg/Day)	% below EAR (500 mg/Day)	% below RDA (700 mg/Day)	% above UL (2500 mg/Day)	% below EAR (2.5 mg/Day)	% below RDA (3 mg/Day)	% above UL (7 mg/Day)
**Total (*n* = 1122)**	0.4 (0.2, 0.6)	26.1 (21.7, 30.4)	0	2.0 (1.3, 2.7)	11.2 (8.7, 13.7)	0	0	0.1 (0, 0.2)	50.8 (45.4, 56.1)
**Sex**									
Male (*n* = 574)	0.5 (0.1, 0.8)	25.0 (18.9, 31.1)	0	2.8 (1.4, 4.2) ^a^	13.1 (8.7, 17.6)	0	0	0.2 (0, 0.3)	46.8 (39.0, 54.6)
Female (*n* = 548)	0.4 (0, 0.7)	26.9 (20.5, 33.3)	0	1.2 (0.6, 1.8) ^b^	9.4 (6.5, 12.4)	0	0	0.1 (0, 0.2)	55.3 (48.2, 62.5)
**Race/ethnicity** ^4^									
Non-Hispanic white (*n* = 332)	0.3 (0, 0.7) ^a,b^	24.3 (18.1, 30.4) ^a^	0	2.1 (1.0, 3.1) ^a,b^	11.5 (7.0, 16.1) ^a,b^	0	0	0 ^a^	48.2 (37.6, 58.9)
Non-Hispanic black (*n* = 261)	0 (0, 0.2) ^a^	18.7 (12.7, 24.8) ^a^	0	3.6 (1.1, 6.1) ^b^	17.4 (10.4, 24.4) ^b^	0	0 (0, 0)	0. 3 (0.1, 0.4) ^b^	51.7 (40.7, 62.6)
Mexican American (*n* = 357)	0.8 (0.2, 1.4) ^b^	36.4 (29.6, 43.1) ^b^	0	1.3 (0.5, 2.2) ^a^	9.6 (6.4, 12.8) ^a^	0	0	0 (0, 0.2) ^a^	50.6 (42.4, 58.9)
**Poverty status**									
Income to poverty ratio <1.85 (*n* = 684)	0.2 (0.1, 0.4)	23.1 (16.7, 29.4)	0	2.3 (1.3, 3.2)	13.8 (10.2, 17.4) ^a^	0	0	0.1 (0, 0.2) ^a,b^	50.0 (43.2, 56.7)
Income to poverty ratio 1.85 to <3.5 (*n* = 204)	0.7 (0.1, 1.4)	32.8 (22.8, 42.8)	0	2.5 (0.8, 4.1)	12.0 (6.9, 17.1) ^a,b^	0 (0, 0)	0	0.2 (0, 0.4) ^a^	53.3 (42.7, 63.8)
Income to poverty ratio ≥3.5 (*n* = 174)	0.5 (0, 1.1)	27.8 (19.5, 36.1)	0	1.8 (0.2, 3.4)	9.0 (4.5, 13.4) ^b^	0 (0, 0)	0	0 ^b^	50.4 (38.0, 62.7)

^1^ Does not include any children who reported consuming breast milk on either day one or day two of the 24 h dietary recall; ^2^ age in months at time of exam in Medical Examination Center; ^3^ since usual intakes cannot be negative, any estimates that were negative (i.e., lower bound for 95% Confidence Interval (CIs)) were truncated at zero and 95% CIs are provided. If a cut-point fell on the distribution of intakes such that no individual was included, these values did not have a standard error; thus, a zero value was given and no 95% CIs were provided; ^4^ race/ethnicity subanalyses are limited to those individuals who report being either non-Hispanic white, non-Hispanic black, and Mexican American; values with superscript letters that differ are significantly different, *p*-value < 0.05; abbreviations: Estimated Average Requirement (EAR), National Health and Nutrition Examination Survey (NHANES), Recommended Dietary Allowance (RDA), Tolerable Upper Intake Level (UL).

## Abbreviations

AAP	American Academy of Pediatrics
B24/P	Birth to 24 Months and Pregnancy
CI	Confidence Interval
EAR	Estimated Average Requirement
FITS	Feeding Infants and Toddler Study
HHS	Health and Human Services
NHANES	National Health and Nutrition Examination Survey
RDA	Recommended Dietary Allowance
UL	Tolerable Upper Intake Level
USDA	United States Department of Agriculture
